# The Timing Statistics of Spontaneous Calcium Release in Cardiac Myocytes

**DOI:** 10.1371/journal.pone.0062967

**Published:** 2013-05-17

**Authors:** Mesfin Asfaw, Enric Alvarez-Lacalle, Yohannes Shiferaw

**Affiliations:** 1 Department of Physics and Astronomy, California State University Northridge, Northridge, California, United States of America; 2 Department de Física Aplicada, Universitat Politècnica de Catalunya, Barcelona, Spain; New Jersey Medical School, University of Medicine and Dentistry of New Jersey, United States of America

## Abstract

A variety of cardiac arrhythmias are initiated by a focal excitation that disrupts the regular beating of the heart. In some cases it is known that these excitations are due to calcium (Ca) release from the sarcoplasmic reticulum (SR) via propagating subcellular Ca waves. However, it is not understood what are the physiological factors that determine the timing of these excitations at both the subcellular and tissue level. In this paper we apply analytic and numerical approaches to determine the timing statistics of spontaneous Ca release (SCR) in a simplified model of a cardiac myocyte. In particular, we compute the mean first passage time (MFPT) to SCR, in the case where SCR is initiated by spontaneous Ca sparks, and demonstrate that this quantity exhibits either an algebraic or exponential dependence on system parameters. Based on this analysis we identify the necessary requirements so that SCR occurs on a time scale comparable to the cardiac cycle. Finally, we study how SCR is synchronized across many cells in cardiac tissue, and identify a quantitative measure that determines the relative timing of SCR in an ensemble of cells. Using this approach we identify the physiological conditions so that cell-to-cell variations in the timing of SCR is small compared to the typical duration of an SCR event. We argue further that under these conditions inward currents due to SCR can summate and generate arrhythmogenic triggered excitations in cardiac tissue.

## Introduction

It is generally believed that sudden cardiac death is induced by a focal excitation that can propagate and form wave break and reentry [1]–[5]. However, the underlying mechanism and the properties of these focal excitations are not well understood. In particular, it is not known what are the factors that determine when a focal excitation will occur in a region of cardiac tissue. This question is important since the propensity for arrhythmia initiation is dependent on the timing of these excitations. In general we expect a focal excitation to be potentially dangerous if it occurs during the diastolic interval (DI) when cardiac tissue is excitable. In this period cardiac tissue can sustain electrical wave propagation which can form wave break by collisions with the wave back of the previous beat, or at anatomical obstacles in the tissue [6].

Much of the work to identify the mechanism for focal excitations has focused on the role of subcellular Ca cycling [7]–[9]. Ca cycling is the process wherein membrane voltage gated Ca channels (LCC) induce Ca release due to Ryanodine Receptor (RyR) channels which control the flow of Ca sequestered in the sarcoplasmic reticulum (SR). The signaling between LCC and RyR channels occurs within submicron scale junctions where a few LCC channels are in close proximity with a cluster of Ca sensitive RyR channels [10]. In this paper we will refer to these junctions as Ca release units (CRUs). Now, under normal conditions RyR channels respond to changes in local Ca concentration due to openings of the local LCC. This coupling occurs because RyR channels are Ca sensitive, with an open probability which increases with the Ca concentration within the CRU. Hence, an LCC channel opening can induce a few nearby RyR channels to open which in turn leads to a large autocatalytic release of Ca from the local cluster. The corresponding large release of Ca from the RyR cluster is referred to as a Ca spark [10], [11]. However, under abnormal conditions, such as Ca overload, RyR channel fluctuations can lead to “spontaneous” Ca sparks, which occur independently of LCC channel openings and consequently the membrane voltage. Since the cell is composed of roughly 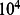 CRUs these spontaneous sparks can induce neighboring CRUs to fire which can lead to a Ca wave which can propagate across the entire cell [7], [12], [13]. The release of Ca due to these subcellular waves is referred to as spontaneous Ca release (SCR). Finally, the large amount of Ca released into the cell due to these waves is pumped out of the cell via the electrogenic sodium-calcium exchanger which induces a net inward current which can depolarize the cell membrane [11], [14], [15]. It is these depolarization events in a population of cells in tissue which induce Ca-mediated focal activity in cardiac tissue.

Ca mediated focal excitations in the heart can only occur if a substantial fraction of cells in tissue exhibit SCR at roughly the same time. This is because SCR induced inward currents must summate across many cells in order to overcome the electrotonic load of quiescent neighboring cells. Thus, to understand how focal excitations are formed it is crucial to characterize the timing statistics of SCR within a cardiac cell, and also across an ensemble of cells in tissue. To date, several experimental and simulation studies have explored the timing of SCR. In particular Falcke et al. [16], [17] applied computer simulation studies showing that SCR is dictated by stochastic wave nucleation events. Also, Ramay et al. [18] showed that SCR timing is sensitive to a variety of factors such as the SR load and RyR gating properties. However, several important questions remain unanswered. Namely, it is not understood how the timing statistics of SCR at the cell and tissue level is related to local properties at the scale of RyR clusters. This question is particularly difficult to answer since it is a multi-scale problem involving a wide range of space and time scales. In this paper we will apply numerical and analytic approaches to address these questions. Our approach builds on the work of several authors, in particular Hinch [19] and Thul et al. [20] (see also [17], [21]–[26] for similar approaches), who have applied the theory of stochastic processes to describe subcellular Ca signaling. As a starting point we will first determine the timing statistics of spontaneous Ca sparks within a single isolated CRU at a fixed SR Ca load. Following this analysis, we will then study the timing statistics of an ensemble of heterogeneous CRUs, with the aim to characterize the first passage time (or waiting time) distribution for spontaneous Ca sparks within a simplified representation of a cardiac cell. While the model considered here substantially simplifies the complex spatial arrangement of Ca release units, our analysis provides a first step to understand the factors that govern the timing statistics of SCR.

## Methods

### The model

**Figure 1:**
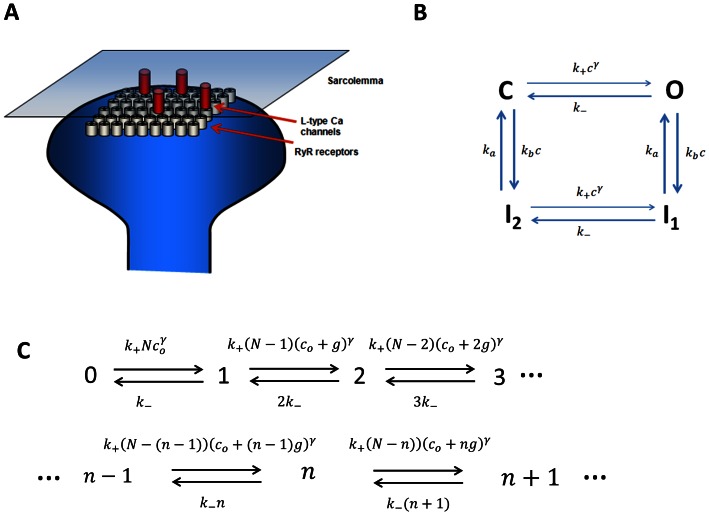
(A) Schematic illustration of the calcium release unit (CRU) showing a cluster of RyR channels on the SR in the vicinity of a few LCC channels on the membrane. (B) Four state Markovian scheme describing the RyR channel. (C) Birth-death process describing the closed to open transitions of 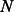 RyR channels in the cluster.

A cardiac cell is composed of roughly 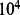 CRUs which are spatially distributed in the cell. A typical CRU is roughly a cylindrical pill box of height 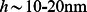 and diameter 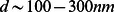 which contains a cluster of RyR channels and a few LCC channels in close proximity (see Fig. (1A)). Experimental studies have shown that the number of RyR channels within clusters is highly variable and range from 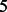 to 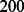 channels, with a distribution that is roughly exponential [27]. Also, Ca diffusion within the CRU is in the range 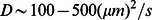 [28], [29], so that the diffusion time across a CRU is 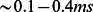, which is faster than the typical RyR channel transitions times 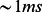. Thus, we can make the rapid diffusion approximation and assume that Ca is spatially uniform within the CRU with a concentration 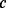 that is 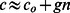 where 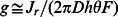. Here, 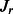 is the Ca flux due to an open RyR channel in units of 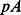, 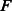 is Faraday's constant, 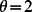 is the charge of the Ca ion, 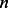 is the number of open RyR channels, and where 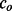 is the diastolic Ca concentration outside the CRU (the cytosol). Physiological parameters used in this study are given in Table 1. Note here that typically 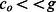 since the diastolic Ca concentration is much smaller than the Ca concentration 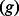 in the CRU when one RyR channel is open 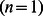. However, the single channel RyR flux 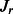 is difficult to measure experimentally and a wide range of values have been reported in the literature [11]. For example several experimental groups have reported a single channel RyR flux of 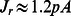 [30], while others have argued that 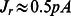 or lower under physiological conditions [31], [32]. Also some studies have argued that the current may be as low as 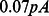 [33]. Given this uncertainty we will treat this quantity as a free parameter in our model, and discuss the properties of the system for a wide range of current amplitudes.

**Table 1:** Model Parameters.

Parameter	Description	Value
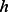	Height of CRU	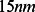
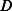	Ca diffusion coefficient	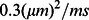
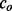	Background diastolic Ca concentration	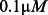
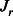	Single RyR channel conductance	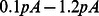
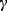	Number of RyR Ca binding sites	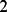
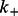	RyR opening rate	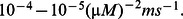
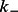	RyR closing rate	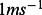
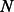	Number of channels in cluster	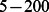

To model the dynamics of RyR channels in the cluster we will consider the reaction scheme shown in Fig. (1B), which is used by Shannon et al. [34]. This scheme has the advantage that it is the simplest Markov state model which describes many important features of RyR channels. The key step in the Markov chain which governs the timing of spark activation is the transition between the closed 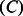 and open states 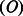, which is dictated by a Ca dependent transition rate. This scheme is used to model Ca-induced-Ca-release (CICR) and is generic to wide variety of Markovian models describing RyR. In this study we will fix 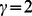 in order to simplify our computations, although all computations presented here can be generalized to an arbitrary power 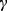. Note here that we have simplified the formulation of Shannon et al. who had included an SR load dependence to the rate 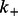. Here, we take 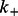 to be constant since we will consider the case of fixed SR load. In the discussion we will relax this assumption and address the case of variable SR load, where 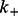 is likely to vary with time. In this study we will follow Restrepo et al. [29] and chose 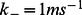 so that the mean open time of an RyR channel is 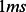. Also, since RyR transition rates are not well established we will take 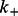 over a broad range 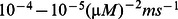 which includes the parameters used in the Restrepo et al. and Shannon et al. studies. In this study we will not fix the inactivation and recovery from inactivation rates 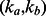, since, as we will argue in the next section, at fixed SR load, these quantities only influence the timing statistics of SCR via the number of RyR channels in the closed state. Hence, we will discuss the qualitative role of these states on spark timing but will not explicitly simulate their dynamics.

## Results

### Statistics of a single CRU

In this section we explore in detail the factors that determine the timing statistics of spontaneous Ca sparks at a single CRU with 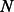 RyR channels. As a starting point we will consider the case where the Ca concentration in the dyadic cleft 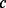 is small so that 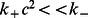 and 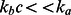. If we wait for times longer than 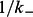 and 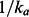 then most of the channels will be in the closed state and spark activation will be dominated by transitions to the open state. In a later section we will relax this assumption and discuss the scenario where the time scale of recovery from inactivation 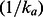 is sufficiently large to influence spark activation. To proceed, we define a spontaneous Ca spark as a fluctuation in the number of open channels, which we denote by 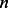, such that 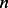 exceeds a critical value 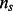. Thus, the instant the number of open channels crosses 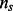 then a spark is said to have occurred at that CRU. Given this criterion then the timing statistics of a spontaneous Ca spark is determined by the probability 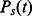 that 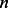 reaches 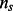, for the first time, within a time interval 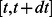. This probability is referred to as the first passage time distribution (FPD), or alternatively the waiting time distribution. To compute the FPD we will make the simplifying assumption that spark activation is dictated primarily by the 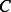 to 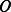 transitions, so that transition rates to the inactivation states are negligible i.e. 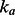 and 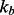 in the full Markov scheme are small. Later, we will relax this assumption and discuss the scenario when this assumption cannot be made. The stochastic dynamics of the cluster is then governed by 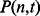 which is the probability that 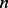 channels in the cluster are open at time 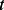. This quantity obeys a Master equation
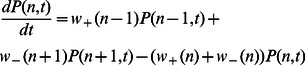
(1)where
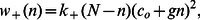
(2)

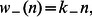
(3)which describes a nonlinear birth-death process (illustrated in Fig. (1C)) between 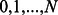 possible states of the cluster. Using this Master equation we will explore the cluster properties in detail with the goal to determine the FPD.

#### Equilibrium points and the effective potential

**Figure 2:**
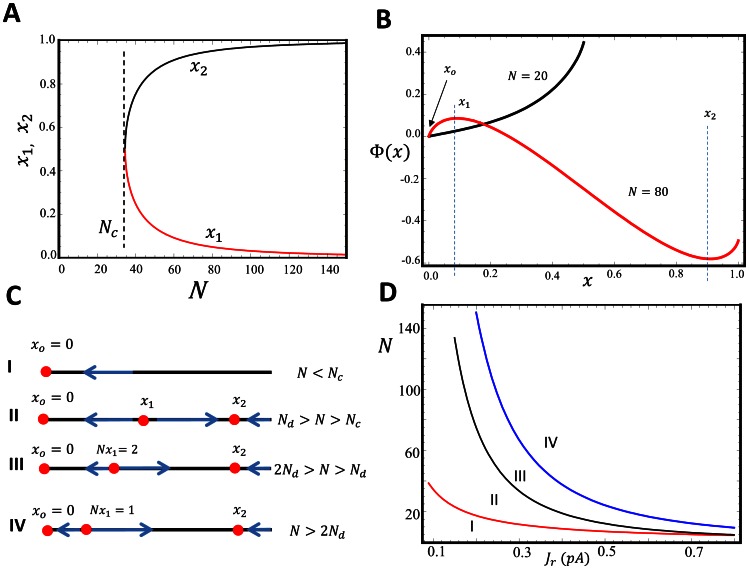
(A) Plot of location of fixed points 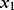 and 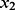 as a function of cluster size 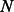. Parameters used are shown in Table 1, where we have fixed 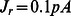, and 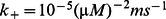. The system exhibits bistability for cluster sizes that exceed 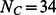 channels. (B) The plot for the effective potential 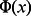 as a function of 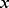. The black and red lines denote the plot for the cluster size 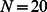 and 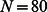, respectively. For the case 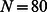, positions 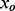 and 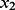 denote the location of local minima, and 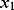 is a maxima. (C) Illustration of fixed points corresponding to the four distinct scenarios. (D) Plot of demarcation lines, in the 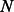 vs 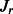 plane, separating the four different fixed point scenarios.

In order to understand the properties of our nonlinear birth-death process describing the RyR cluster, it is first necessary to determine the probability flow on our discrete state space. As a starting point let us first consider the large 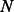 limit, where the system can be conveniently mapped to a continuum. To proceed, we follow Hinch [19] and first define the fraction of channels in the open state as 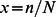, and the functions 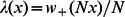 and 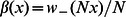. Applying detailed balance between discrete sites gives, in the large 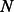 limit, an equilibrium distribution
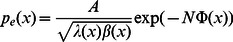
(4)where 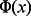 is an effective potential
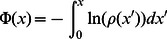
(5)with 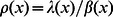, and where 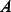 is normalization constant. The effective potential allows us to identify fixed points, that satisfy 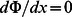, which will dictate the stochastic evolution of the system. In particular, minima of the effective potential serve as metastable states in the sense that the trajectory of 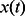 will tend to fluctuate around these points. These stationary points are given by the condition 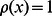, which reads
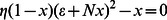
(6)where we have introduced the dimensionless parameters 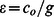 and 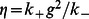. In the physiological range (see Table 1) solutions to this equation can be approximated as follows: (i) There is a real solution at 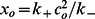 valid for all physiological cluster sizes 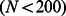. (ii) Two real solutions 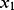 and 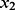 for cluster sizes larger than 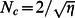. These solutions are well approximated by
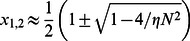
(7)which for large clusters, where 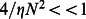, can be approximated more simply as 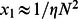 and 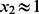. A straightforward analysis of these fixed points shows that that 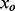 and 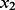 correspond to local minima of the effective potential, while 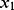 is always a local maximum. We note here that the cluster size 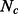 will feature prominently in the subsequent discussion as it determines the onset of bistability of the RyR cluster. In Fig. (2A) we plot the stationary points 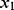 and 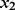 as a function of system size 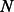, showing the emergence of two real roots once the cluster size exceeds 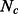. For the parameters used in this simulation 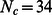 channels. In Fig. (2B) we plot the effective potential for two cluster sizes displaying mono and bistability. In the bistable regime the closed stable state at 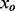 is separated from the fully open cluster at 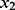 by a potential maxima at 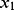.

#### Discrete channel transitions

The above analysis applies in the large 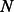 limit as long as the location of the stationary points can be well approximated using the continuum approximation. In particular, this approximation only applies providing 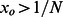, and if the difference between the first two stationary points satisfies 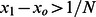. Applying the parameters from Table 1, we find that 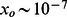 which is far less than 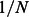 for cluster sizes in the physiological range 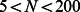. Hence, to understand the dynamics of the cluster it is crucial to evaluate the discrete channel transition rates near the shut state 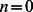. To analyze the discrete dynamics we consider the bond between sites 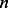 and 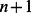 and define the ratio
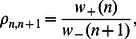
(8)which is simply the discrete counterpart to the function 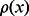. Thus, if 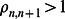 then there are more transitions from the 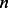 to 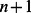 state than vice versa. Similarly if 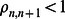 then the probability flux is in the opposite direction. Thus, if there exists a site 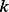 where 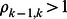 and 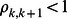 then this site defines a local equilibrium on the discrete lattice of sites. To proceed, we evaluate 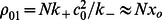, and find that for the range of physiological parameters 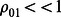. Thus, on our discrete lattice 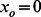 is always a stable stationary point i.e. the shut state of the cluster is always stable.

The continuum approximation thus holds providing 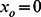 and with two fixed points 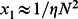 and 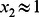 with the additional constraint that 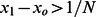. However, this requirement breaks down for cluster sizes 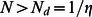. Thus, if the RyR cluster exceeds a critical size 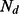 then it is necessary to analyze the discrete channel transition rates. To proceed we evaluate the ratio of rates for the next two states of the Markov chain which yields
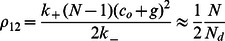
(9)

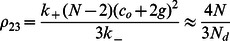
(10)where the approximation is valid when 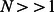 and 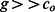. Therefore, if 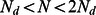 then the 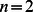 Markov state is unstable in the sense that the probability flux flows towards 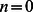 if the system starts at 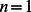, and flows towards the fully open state at 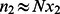 for any 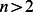. Similarly, if 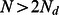 then the 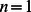 state is unstable in the sense that for any 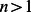 the probability flux is towards the fully open cluster state. Therefore, for 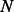 in this range we need only one RyR channel to open to induce a full cluster opening. The structure of fixed points of our RyR cluster is illustrated in Fig. (2C) showing the four distinct cases that can occur, as a function of cluster size. In Fig. (2D) we plot the RyR cluster sizes 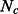, 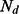 and 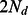 as a function of the single channel RyR current 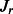. These lines demarcate the cluster sizes which display monostability, bistability, and also the discrete bistable regime dictated by one and two channel openings.

### The mean first passage time for a cluster of RyR channels

Once we have characterized the equilibrium points on our lattice we seek to determine the statistics of spontaneous Ca spark activation. Let us first compute the MFPT mean-first-passage-time (MFPT) from the 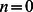 state to 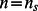, where 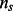 is our criterion for a Ca spark. To choose the spark criterion 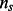 we note that in the bistable regime 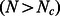 the criterion for a spark is clearly that the number of open channels crosses the effective potential barrier at 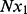 i.e. our cluster should fully open in order to release a substantial amount of Ca into the cell. Therefore, in this regime our spark criterion is simply that 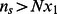. A convenient feature of our system is that in the bistable regime the midpoint of our lattice 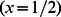 is always larger than the location of our potential barrier maximum at 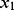. Therefore, the MFPT to 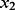 will be essentially the same as the time to 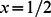 since the MFPT is dominated by the time to reach the potential maxima at 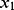, since for 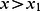 the effective potential is downhill. Thus, a convenient criteria for the timing of a Ca spark is to pick 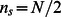. Now, if the cluster is not bistable 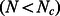 then only the shut state is stable and the system will have to climb uphill to reach 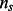. In this case the MFPT will be sensitive to the chosen spark criteria 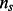. To compute the MFPT we follow Pury et al. [35] who have computed an exact expression for the MFPT to go from an initial state of 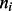 open channels to a final state 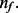 This expression is given by
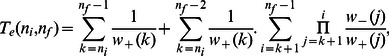
(11)which is valid in the case of reflecting boundary conditions imposed at 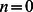. In our study we will fix 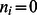 and 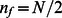, for 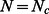, and 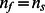 for 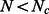. However, Eq. (11) does not give insight into the parameter dependence of 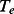. To gain further insight we will follow Doering et al. [36] and proceed to evaluate the large 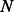 behavior of this expression. Our main results are summarized bellow. All details of the computations are given in Appendix S1.

#### Case I

Clusters of size 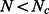. In this case there is only one stable fixed point at 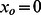, and a Ca spark occurs when the fraction of open channels reaches 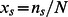. In the large 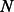 limit we find that to leading order the MFPT has the form 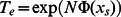, where 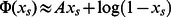, and where 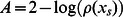, with 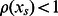. Thus, in this regime we have that 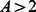 and 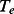 increases exponentially with 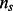 the number of channels necessary for a Ca spark. So that even for a relatively small criterion for 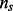, say 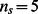 channels, then 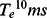, which is orders of magnitude larger than the cardiac cycle 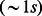. Therefore, we do not expect clusters in the monostable regime 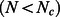 to contribute to the timing of SCR in a cardiac cell.

#### Case II

Clusters of size 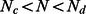. In this case our cluster is bistable and the continuum approximation is valid. Our final result is that in this regime the MFPT can be well approximated using
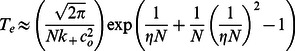
(12)


#### Case III & IV

Clusters of size 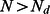. In this case the MFPT is dominated by the transition time from the shut state of the cluster to two open RyR channels. The MFPT for this discrete transition can be well approximated as
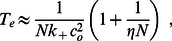
(13)which is valid for 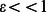. For 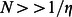 the MFPT is well approximated by the first term in Eq. (13)

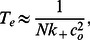
(14)which is simply the MFPT for a single RyR channel opening in the cluster.

**Figure 3:**
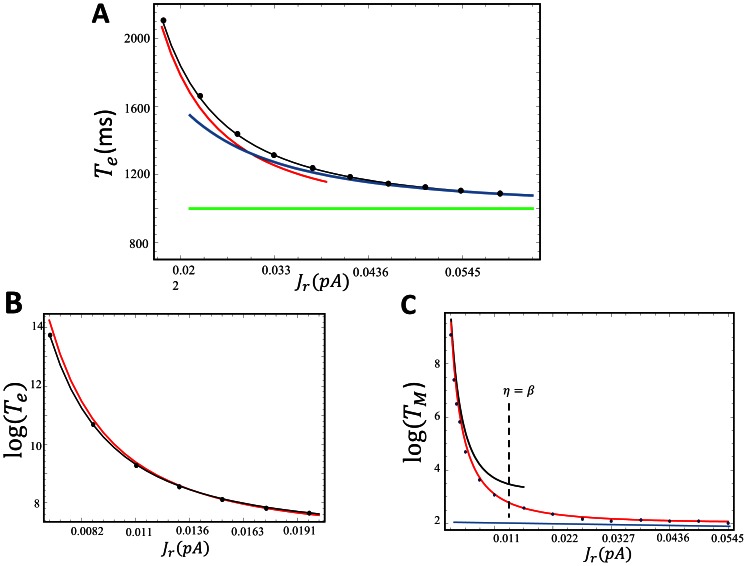
(A) Plot of 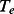 vs the RyR single channel current 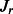. The curves shown are numerical solution using the exact stochastic algorithm (black circles), exact solution (black line) according to Eq. (11), asymptotic solution valid in regime II Eq. (12) (red line), high excitability limit using Eq. 13 (blue line), and finally the MFPT for a single channel opening given by Eq. (14) (green line). Here, we fix 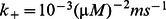 and 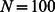. (B) Plot of 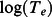 vs 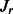 for small 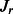 for the same parameter choice as Fig. (3A). Black circles are numerical simulation results, black line is the exact solution using Eq. (11) and the red line is the asymptotic solution given by Eq. (12). (C) Plot of 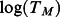 vs 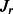 using 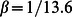 and 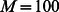. To speed up simulations we have used 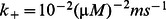. Black circles are the numerical simulation results, the red line is computed using the summation shown in Eq. (24). Black line is the asymptotic approximation evaluated via Eq. (27) and the horizontal blue line is the high excitability limit given by Eq. (23). The vertical dashed line indicates the current 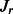 where 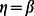.

These analytic results provide a complete picture of the MFPT to a spontaneous Ca spark in an RyR cluster. In particular we note that the parameter 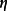 controls the exponential dependence of 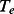, and that the ratio of 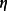 to 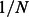 determines the crossover from exponential to algebraic behavior. Hereafter, we will refer to 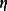 as the “excitability” of the cluster as it will feature prominently in our subsequent analysis. To evaluate the validity of our results we have implemented a standard Gillespie algorithm [37] to simulate the stochastic dynamics of the Markov chain given in Eq. (1). In these simulations we computed the time for the cluster to transition from 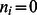 to 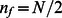, and computed the MFPT by averaging over 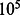 independent stochastic trajectories. In order to speed up our computations, which can be exceedingly long for low 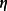, we have used an RyR open rate of 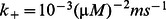 which is larger than the physiological range shown in Table 1. In Fig. (3A) we compute 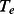 vs the RyR current flux 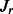 for a fixed cluster of size 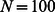. On the same graph we show results of the stochastic simulation, the asymptotic expression given by Eq. (12), along with Eqs. (13) and (14). To further confirm the asymptotic predictions in Fig. (3B) we plot 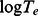 vs 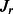 showing excellent agreement across four orders of magnitude.

### First passage time Statistics of an ensemble of junctions

A cardiac cell, under appropriate conditions, can exhibit Ca waves which can propagate across the cell. When this occurs a large amount of Ca is released into the cell and this phenomenon is referred to as spontaneous Ca release (SCR) [38]–[41]. It is generally believed that spontaneous Ca waves are nucleated in regions of the cell where spontaneous Ca sparks occur, which release enough Ca to stimulate nearest neighbor CRUs via a fire-diffuse-fire mechanism [16], [42]. This result is supported by optical mapping experiments showing that SCR in cardiac cells are due to waves that originate in localized regions well bellow the mapping resolution [14], [43]. Based on these observations we will make the simplifying assumption that the timing of wave nucleation is determined by spontaneous Ca sparks that occur at a subset of CRUs in the cell. These nucleation sites are likely to be in regions of the cell where there is a larger than average number of RyR clusters, so that a spontaneous Ca spark is likely to nucleate a Ca wave in that region. Thus, we will assume that there are 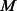 possible nucleation sites in the cell, where 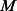 is dependent on the SR load and also the spatial distribution of RyR clusters in the cell. We stress here that this is an approximation since wave nucleation is likely to be a complex process involving cooperativity between spatially distributed junctions in the cell. Our key assumption here is that once a spontaneous Ca spark occurs at a nucleation site then a Ca wave will develop with very high probability i.e. the key stochastic event is the first Ca spark at that site. This assumption should apply at sufficiently high SR loads where a spontaneous Ca spark will release enough Ca to initiate a Ca wave.

To proceed let us assume that we have 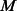 CRUs in the cell where wave nucleation can occur, and that 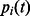 is the FPD for a spontaneous Ca spark at the 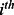 CRU. Then the probability that one of these 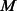 junctions will fire for the first time in the time interval 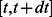 is given by 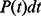 where
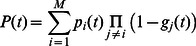
(15)and where
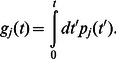
(16)


In general an analytic expression for 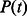 is difficult to compute since the single junction FPD 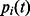 is itself difficult to determine. However, we note that there are two important limits in our problem where the distributions 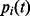 can be well approximated as a single exponential, which allows Eq. (15) to be evaluated directly. For a general computational approach which accounts for the detailed structure of the RyR cluster, and which can be applied to more complex Markovian models, see Ref. [26]. To proceed, we follow Lindenberg et al. [44] who show that in the high barrier regime, which corresponds to case I & II here, then the FPD can be well approximated by an exponential distribution.
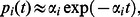
(17)where 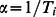 is the inverse of the MFPT for the 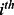 CRU. This approximation rests on the observation that if the MFPT of the 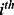 CRU 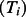 is much larger than temporal correlations in the open probability trajectory 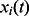, then the timing of spontaneous sparks are, to a good approximation, statistically independent. In this scenario the FPD is exponentially distributed since the escape rate is then effectively constant. This approximation applies in case I & II since in this regime barrier hoping is rare, and occurs on a time scale much larger than the local dynamics near the stable shut state. Now when the high barrier approximation is no longer valid then Eq. (17) cannot be derived on the basis of statistical independence. However, in regime IV, where the timing is dictated by a discrete channel transition to one open channel, then the exponential form in Eq. (17) still holds since the closed time of RyR channels is exponentially distributed. Thus, we can apply the approximation in Eq. (17) providing the clusters in question are in regions I,II, and IV. In these cases we have
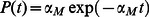
(18)where 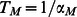 is the MFPT of 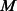 junctions and is determined by
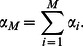
(19)


The expression above allows us to compute the MFPT for an ensemble of 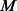 junctions in the cell. Here, we will explore the behavior of this quantity under various scenarios. In particular, we will consider separately the case of small 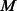 where there are only a few nucleation sites in the cell, and also the case when wave nucleation can occur from a large population of CRUs. As a starting point let us consider the case where there are only a few nucleation sites with channel numbers 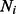 and excitability 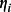, with 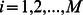. Now, if all clusters are in the exponential regime, and Eq. (12) can be applied, then the sum in Eq. (19) will be dominated by that cluster which minimizes the exponential term. To leading order this will be that cluster with the maximum 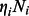, which is the product of excitability and channel number. Thus, in this regime the MFPT to SCR will be dictated by a single nucleation site since that site will have an MFPT which is exponentially smaller than the other CRUs. Now, for larger SR load we will consider the case where the timing of spontaneous release is given by Eq. (14). In this scenario we can easily compute the MFPT to SCR as 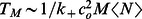 where 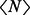 is the average number of channels in the 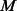 clusters. Note here that in this regime the MFPT is reduced by a factor 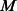 since any one of the 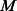 clusters can fire first. Also, since the MFPT of all the 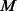 clusters is comparable then SCR can potentially be nucleated at each site. This is in contrast to the exponential regime where SCR is likely to be dictated by a single cluster.

Now, in the case where 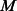 is large then it is necessary to explore the behavior of the MFPT where the size and properties of each CRU is determined by a realistic probability distribution. Here, we will follow Badeley et al. [27] who have measured the distribution of RyR cluster sizes in a rat ventricular myocyte. The key finding there is that the number density of clusters with 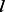 channels, which we denote here as 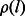, is given by an exponential distribution
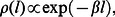
(20)where 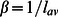, where 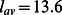 is the average number of channels per cluster in the cell. This finding indicates that the distribution of RyR channels is broad and therefore variations in channel number are likely to determine the timing statistics. Here, we will compute analytically the MFPT for an ensemble of junctions that are exponentially distributed. To proceed, we write Eq. (19) as
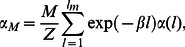
(21)where 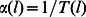 where 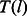 is the MFPT for a cluster with 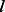 channels. Here, we have set 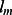 to be the size of the largest cluster in the cell, and where 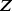 is a normalization factor defined by
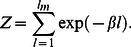
(22)


A direct evaluation of this normalization factor yields 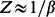, providing 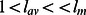. To compute the MFPT for our system we first consider the large 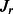 limit when all clusters are in region IV of the discrete bistable regime. In this scenario the FPD is exponential and we can compute 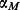 by directly evaluating Eq. (21) using 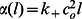. This computation is straight forward and yields a MFPT of
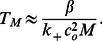
(23)


Now as 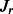 is decreased then a significant fraction of clusters will be in Region II. In this regime we apply Eq. (12) and evaluate sum
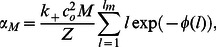
(24)where
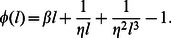
(25)


To estimate 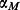 we note that the sums will be dominated by the minima of 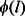 which occurs at cluster sizes
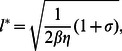
(26)where 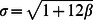. To proceed, we can approximate the sum in Eq. (24) using a saddle point approximation which yields a compact expression
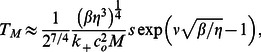
(27)where we have applied the condition 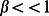, and where 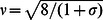, and 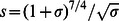. Here, we note that this result is only valid providing the dominant clusters of size 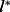 is in the bistable regime (region II) i.e. 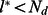 which is approximately equivalent to the requirement that 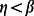. Also, it is important to note that Eq. (27) does not apply when 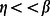 since the system is no longer bistable and therefore Eq. (25) cannot be applied. However, in this case the MFPT is exponentially large and we do not expect SCR to occur on a time scale comparable to the cardiac cycle. Therefore, Eq. (27) captures the correct crossover from algebraic to exponential timing in the case where the cluster number is exponentially distributed.

In order to confirm the analytic predictions we directly simulate the exact stochastic evolution of 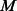 independent clusters with a number of channels that are taken from an exponential distribution. In Fig. (3C) we plot 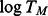 vs 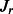 using the exact stochastic simulation, along with the analytic predictions given by Eqs. (23) and (27). Note here that to speed up our computations we have used a forward rate that is an order of magnitude larger than the physiological value. As shown, for large 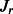, the MFPT converges to a constant that is determined exactly by Eq. (23). Furthermore, as 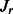 is decreased 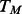 rises substantially and is well approximated by the asymptotic expression given by Eq. (27). Also, included is the exact summation of Eq. (21), which matches almost exactly with the numerical simulations. This result confirms the validity of the exponential approximation given in Eq. (17). To summarize, we identify a crossover from exponential to algebraic dependence of the MFPT as a function of the excitability parameter 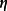. For high excitability 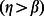 the MFPT can be approximated by Eq. (23), and as the excitability is lowered so that 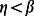 the MFPT grows exponentially as predicted by Eq. (27). To predict the condition for this crossover we note that 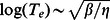 so that the exponential rise in the MFPT occurs roughly when the excitability is 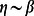. In Fig. (3C) we indicate the value of 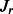 such that 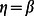 (vertical dashed line) which correctly predicts the crossover region. This result gives an qualitative estimate of the degree of excitability bellow which SCR becomes exponentially rare.

### Statistics of spontaneous Ca sparks under varying SR load

**Figure 4:**
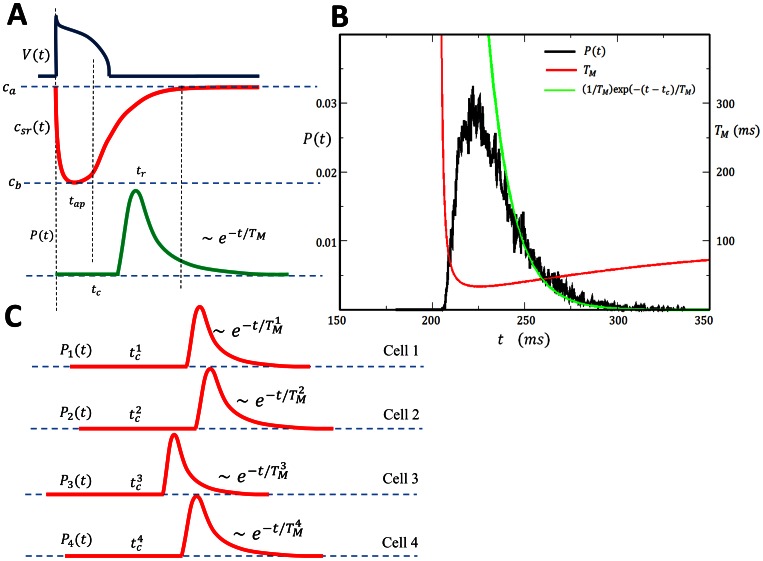
(A) Schematic illustration of the voltage time course, SR Ca concentration, and the FPD, following an AP. (B) Plot of 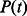 (black line) computed using the exact stochastic algorithm with time dependent excitability according to Eq. (29). Probability distribution is computed by binning the first passage time of 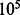 independent samples. The parameters used are 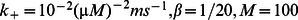. Red line corresponds to a plot of 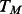 using Eq. (27). The units of 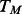 is indicated on the right y-axis. Late time behavior of 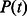 is fitted using an exponential with decay rate 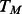 (green line). (C) Schematic illustration showing cell-to-cell variations of the FPD 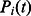.

In this paper we have characterized the timing statistics of spontaneous Ca sparks in an ensemble of heterogeneous junctions. A key assumption that we have made throughout, is that system parameters, such as the SR Ca concentration 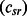, are constant and independent in time. Thus, our results can only be applied to a quiescent cardiac cell in which physiological parameters have reached their steady state values. However, cardiac cells in the heart are typically driven by an AP and the SR load changes substantially as a function of time. In this section, we will analyze the first passage time distribution of spontaneous Ca sparks after a cell has been stimulated by an AP. Recall, that following an AP, Ca is released from the SR due to Ca sparks that are triggered by voltage gated LCC Ca channel openings. Thus, the SR load is substantially depleted and then gradually recovers as Ca is pumped back into the SR via SERCA pumps. In Fig. (4A) we illustrate a typical response of the SR Ca concentration as a function of time. Here, the cell is driven by the AP shown (blue line), and the SR concentration (red line) is depleted from its initial value denoted as 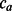. This depletion time is relatively fast 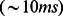
and we expect the SR to remain depleted, at concentration 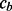, for a duration 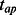 which is determined by the time course of LCC inactivation. Once the voltage returns to the resting membrane potential the LCCs are shut and the SR load recovers back to 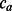 over a time scale 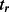. Given this setup we will now characterize the shape of the first passage time distribution (FPD), which we denote here by 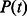, following the AP. For simplicity, we will consider conditions where the initial SR load is high so that at fixed 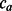 the system is in the discrete bistable regime (region IV). We will also assume that at the depleted concentration 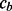 spontaneous sparks are rare and the MFPT is exponentially large i.e. the system is in region I & II. To determine the shape of the FPD under varying SR load we will apply Eq. (27) which describes the crossover from exponential to algebraic behavior as a function of the excitability parameter 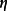. We note that 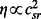 so that for low SR loads 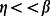 and 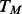 is exponentially large. Thus, in the time dependent case we expect that 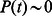 for small 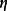 i.e. the system will be effectively refractory. Now as the excitability 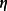 increases, as the SR load rises, then 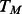 will decrease rapidly due to the exponential sensitivity 
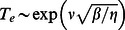
. Therefore, we expect 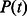 (Fig. (4A) green line) to increase substantially once the SR load has reached a level such that 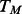 is of the order of the cardiac cycle 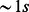. Now, as the SR load increases further then 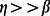 and 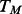 is well approximated using Eq. (23) and is independent of SR load. In this regime the FPD is well approximated as an exponential with decay rate 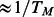 Hence, our final result is that we can approximate the FPD for a cardiac cell, following an AP, as a shifted exponential of the form
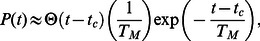
(28)where 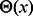 is the Heaviside step function. The MFPT of this distribution is then 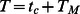, so that the effect of SR depletion is simply to increase the waiting time by the refractory period 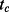. Interestingly, a phenomenological first passage time distribution of the form given in Eq. (28) has been used to describe 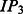 -evoked Ca spikes which govern the timing of Ca oscillations in a variety of cell types [21], [25]. Thus, we expect that the key assumptions underlying Eq. (28), namely the existence of an effective refractory period followed by Poission statistics, should describe general features of Ca wave nucleation phenomenon.

In order to check the validity of our heuristic arguments above we numerically simulate our system of 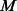 junctions with time dependent parameters. To simulate variable SR load we will vary the model parameter 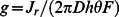, where 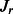 is the single RyR current flux which will vary with SR load. Thus, we will consider a time dependence
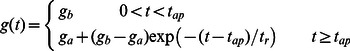
(29)where 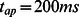, 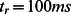, and 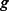 varies from low 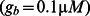 to high 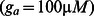 excitability. In Fig. (4B) we show the numerically computed FPD under these conditions showing an effective refractory period, followed by a rapid rise and an exponential decay. On the same graph we plot 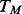 using Eq. (27) showing that the effective refractory period is well approximated by the time at which 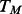 drops to the physiological range i.e. 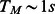. This result confirms our argument that the strong exponential sensitivity of the MFPT on the excitability 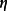 gives the FPD an effective refractory period. Also, we plot an exponential with a decay rate 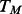 given by Eq. (23) confirming our prediction that the long time behavior of the FPD is exponential.

### The timing distribution of SCR across an ensemble of cells

In this section we will apply our previous analysis to determine how SCR is synchronized across a population of cells in cardiac tissue. This is an important question since in order for SCR to induce a focal excitation in tissue a large fraction of cells must undergo SCR at roughly the same time. Therefore it is crucial to analyze the nature of cell-to-cell fluctuations in the FPD. Our previous analysis reveals that the FPD for a cardiac cell is effectively determined by the refractory time 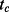 and the spontaneous spark rate at high SR loads 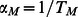. Thus, the relative timing of SCR in cardiac tissue will be crucially dependent on the cell-to-cell fluctuations of these quantities. To analyze these fluctuations we will consider a cardiac tissue with 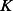 independent cells, and determine the firing time distribution 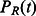 so that 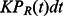 gives the number of cells in our tissue in which SCR occurs for the first time in the interval 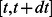. This distribution is given by
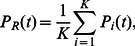
(30)where 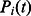 is the FPD for the 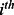 cell. Using this distribution it is straight forward to compute the average firing time in a tissue of 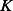 cells which is
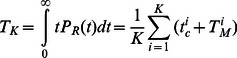
(31)so that 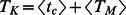, where the brackets denote averages over the ensemble of 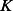 cells in the tissue. To determine the relative timing of SCR in tissue we seek to compute the standard deviation of 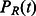 defined as
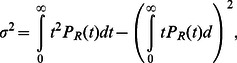
(32)inserting Eq. (28), and assuming statistical independence of 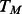 and 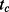 gives
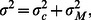
(33)where 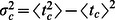 is the variance of the refractory period, and 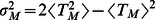 is the variance due to cell-to-cell fluctuations of the MFPT (see Appednix S2 for calculation details). Therefore, the relative timing of SCR in tissue is determined by cell-to-cell variations in the refractory period 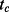 and the MFPT at high SR loads 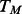. The relevance of these results to the formation of Ca mediated ectopic activity will be addressed in the discussion.

## Discussion

In this paper we have studied the timing statistics of spontaneous Ca sparks at the single CRU, cell, and tissue level. We find that the MFPT to a spontaneous Ca spark in a single CRU is dictated by a dimensionless quantity 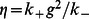 which is a measure of the excitability of the cluster. For low excitability 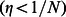, where 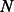 is the number of channels in the cluster, the MFPT is exponentially sensitive to system parameters as given by Eq. (12). In this regime the RyR cluster is bistable and a spontaneous Ca spark occurs when random RyR transitions to the open state are of sufficient number to cross a potential barrier separating the closed and fully open state of the cluster. In this case, RyR clusters display similar statistics to the classic Kramer's barrier crossing problem [45], in which the first passage time is exponentially sensitive to the barrier height. A consequence of this feature is that the frequency of spontaneous Ca sparks is exponentially sensitive to the SR Ca load which is the main dynamical variable which controls the excitability 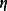 On the other hand, in the high excitability scenario 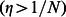 the system is in the discrete bistable regime where only one, or a few, channels need to open to induce a spontaneous Ca spark. In this regime the MFPT is given by Eq. (13), revealing an algebraic rather than exponential dependence on system parameters. This result is not surprising since the timing statistics of spontaneous Ca sparks is now dependent only on the transition rate of a few RyR channels. Consequently, for very large excitability 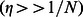 the MFPT is not dependent on the current flux across the RyR channel, and can only depend on the SR load via the RyR opening rate 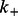.

In a cell with several thousand CRUs then we expect that SCR is dictated by wave nucleation from a population of 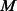 CRUs. These CRUs are those which are located in regions of higher than average RyR channels so that they will serve as wave nucleation sites. In the limit of high SR load the timing of SCR is then given by 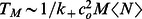 where 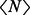 is the average number of channels of the 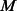 clusters. To estimate this time scale we will use physiological parameters 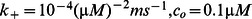 so that 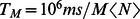. Then 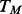 becomes comparable to the cardiac cycle 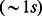 when 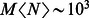. Thus, if the average channel number is large compared to the typical cluster, say 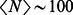, then SCR will occur on the time scale of the cardiac cycle only when the number of nucleation sites reaches 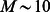. On the other hand, for small clusters to drive wave nucleation 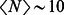, a large number of nucleation sites are necessary 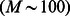. Now, for lower SR load the timing of spontaneous Ca sparks will be exponentially sensitive to system parameters (Eq. (12)). In this regime we expect that one CRU will have a spark rate that is exponentially faster than the other 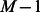 CRUs, and thus, this CRU will dictate the timing statistics of SCR in the cell. However, it should be noted that this conclusion assumes that a Ca spark at the earliest CRU is sufficient to induce SCR. In fact, it is more likely that in this parameter regime of low excitability cooperativity of CRUs will be crucial to initiate SCR. However, these effects are beyond the scope of our analysis which does not account for the coupling between CRUs. Nevertheless, in the simplified setting considered here, our prediction is that at high SR load conditions SCR will originate randomly from many sites in the cell. As the SR load is decreased then the number of sites will decrease until only one or a few CRUs will drive the system. Thus, our findings suggest that the SR load will dictate both the timing statistics and the location of wave initiation sites.

In the case where 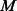 is large and where the number of channels in each CRU is exponentially distributed, then 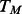 can also be computed analytically. In the low excitability regime 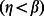 the MFPT is exponentially sensitive to system parameters according to Eq. (27). On the other hand at high excitability 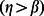 the MFPT displays an algebraic dependence on system parameters according to Eq. (23). Thus, at the whole cell level, measurements of the MFPT should display a distinct crossover as a function of the excitability 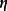. Here, we will evaluate the conditions for this crossover assuming the physiological parameters given in Table 1. Recall that 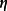 is dependent on the single RyR current flux 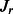, which itself is directly proportional to the SR Ca concentration 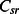. Using these parameters we find a crossover roughly when 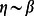 which occurs at a single current flux of 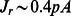 for 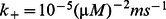, and 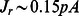 for 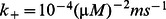. Now, Wang et al. [30] have argued that their experiments indicate a single RyR flux roughly 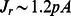, which suggests that for 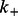 in the range considered here, the system is in the high excitability limit and the MFPT is dictated by a few channel openings. However, if 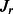 is reduced, perhaps by a reduction in SR load, we expect to observe a crossover to the exponential dependence given by Eq. (27).

The analysis presented in this paper provides a quantitative approach to determine the relative timing of SCR in cardiac tissue. This is an important question to address since in order to depolarize tissue a substantial number of cells must undergo SCR within a time interval that is comparable to the typical duration of an SCR event. Thus, cell-to-cell variations of the timing of SCR are an important factor to determine whether or not SCR can induce a focal excitation in cardiac tissue. Furthermore, it is crucial to analyze this relative timing under physiological conditions where the SR load is depleted following an AP. To address this question we first showed that the FPD to a spontaneous Ca spark, following SR depletion, has two distinct features: a refractory time 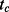, following the AP, where the SR load is depleted to an extent where spontaneous Ca sparks are exponentially rare; and an exponential phase for times 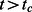 where the FPD decays exponentially with time constant 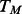. Here, we have assumed that the steady state SR concentration is large, and 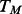 is well described by the high excitability limit given by Eq. (23). We argue that this case is the most physiologically relevant since SCR is typically observed at high SR loads, where the system is likely to be in the high excitability limit. Under these conditions the relative timing of SCR in tissue is then governed by the standard deviation given by Eq. (33). To estimate this quantity it is first necessary to evaluate the cell-to-cell variations of the two time scales 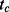 and 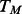. However, given the lack of direct experimental data we will first consider the case where cell-to-cell variations are negligible i.e. we will consider a lower bound to the standard deviation. In this case the variance is given simply by 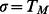, since variations in SCR timing are due solely to the single cell FPD, which is well approximated by an exponential. Now, recall that SCR is initiated by a spontaneous Ca spark that induces a Ca wave in the cell. The lifetime of these waves is in the range 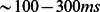, and can vary widely depending on the number of initiation sites and the wave speed. Therefore, if 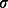 is bellow this range then we expect that SCR in an ensemble of cells will occur with small dispersion and overlap in time. In this case the total inward current generated by SCR in an ensemble of cells in tissue can be substantial and the tissue can be depolarized. To estimate 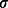 we use our physiological parameters in Table 1 and evaluate 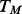 using Eq. (23), which gives 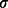 in the range 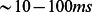, for 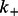 in the range 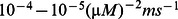. Therefore, for physiological parameters, the variance in the timing of SCR can be much smaller than the duration of SCR itself (see Fig. (4C) for a schematic illustration of this case). In this scenario SCR will be effectively synchronized across a large number of cells in cardiac tissue.

The results in this study, explain to some extent, the experimental observations of Wasserstrom et al. [40] who applied confocal line scan imaging to measure the occurrence of SCR across a group of cells in the intact rat heart. These authors showed that under Ca overload conditions induced by a large external Ca concentration 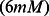, and rapid pacing 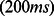, SCR in different cells occurred with remarkably low cell-to-cell variability. An inspection of the line scan images in that paper shows that the typical duration of SCR was roughly 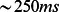, while most SCR events occur within a 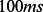 time interval. This data indicates that under these Ca overload conditions the duration of SCR was substantially longer than the timing variability of the occurrence of SCR. Therefore, it is likely that inward currents generated by SCR in a large fraction of cells will summate to form a substantial depolarizing current, most likely via the sodium-calcium exchanger. In fact these authors reported that in one sample the timing of an extra beat coincided precisely with the average time to SCR for cells observed in the mapping field. This result demonstrates that the ectopic beat was likely caused by the near simultaneous occurrence of SCR in a large number of cells in tissue. Our analysis in this paper suggests that in these experiments: (i) The MFPT is described by Eq. (23) and is small relative to the duration of SCR i.e. 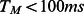. (ii) The cell-to-cell variability of the refractory time 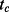 is also small and is not sufficient to desynchronize the timing of SCR. In effect, in these cells SCR occurs nearly simultaneously once the SR concentration reaches the threshold of excitability at roughly the same time 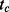. This experimental study, along with our analysis in this paper, highlights the crucial importance of cell-to-cell variability of SCR in order to gauge the propensity for Ca-mediated triggered activity in cardiac tissue.

Our analysis of SCR timing statistics assumed that spark activation is dictated primarily by the 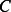 to 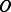 transition rates of the RyR channel. Here, we discuss qualitatively the role inactivation and recovery from inactivation. Firstly, we consider the case where inactivation is strong so that the parameter 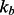 in our Markov scheme shown in Fig. (1B) is large. In this case we expect that the maximum number of open RyR channels during a spark will be small since RyR channels quickly inactivate once they open. This scenario is consistent with the experimental data presented by Wang et al. [30] who claimed that a Ca spark is likely due to the opening of only 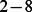 RyR channels in a cluster. In this study they also estimated the single RyR current flux to be 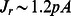, so that only a single channel openning raises the Ca concentration to 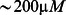. Here, the single RyR flux is necessarily large since only a few RyR openings summate to form a Ca spark which is known to have a peak flux in the range 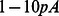. Now since the single RyR flux is large then it is likely that spark activation is dictated by the statistics of the first few channel openings. Therefore, in this scenario we expect that the MFPT will be well described by our discrete bistable regime i.e. regions III & IV, and Eq. (23) for an ensemble of clusters, since inactivation does not play a role in the statistics of first openings. A second issue to consider is the role of recovery from inactivation, governed by the parameter 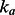 in our RyR Markov scheme. If this parameter is small, so that the time scale 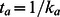 is comparable to 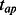 and 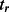 then recovery from inactivation will play an important role to determine the effective refractory time 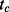. In this case, the number of available RyR channels in the cluster, which can transition to the open state, will be time dependent and recover with a time scale governed by 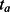. Thus, to determine the timing statistics it will be necessary to replace the variable 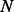 with 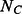 the number of RyR channels in the closed stated. Therefore, if 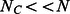 then the MFPT will be substantially modified, and recovery from inactivation will be the main process that dictates the timing of SCR. This scenario is potentially important and is worth further investigation.

An important limitation of our analysis is that we have little experimental data to assess the magnitude of cell-to-cell variations in the refractory period 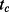. In general, we expect 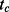 to vary between cells due to variations in the time course of the SR Ca concentration. These variations can be attributed to cell-to-cell fluctuations in ion channel density, especially of the SERCA pump which is the main current responsible for SR Ca reuptake. Our study shows that these fluctuations will have to be measured in order to fully quantify the relative timing of SCR in tissue. A further limitation of our study is that we have assumed that the timing of wave nucleation is determined only by the formation of spontaneous Ca sparks. A more realistic picture should involve the cooperative dynamics of several clusters which are in proximity in regions of the cell with a higher than average number of RyR channels. However, a detailed analysis of this effect will require a more complete description of Ca wave nucleation within a random three dimensional distribution of channels. Nevertheless, our analysis should lay the ground work for a more complete understanding of the timing statistics of wave nucleation in heterogeneous environments.

## Supporting Information

Appendix S1 **The large N approximation to the exact mean first passage time given by** **Eq. 11** **.** Detailed derivations of Eqs 12–14 are given.
(TEX)

Appendix S2 **Analysis of the variance of the first passage time distribution.** A detailed derivation of Eq. 33 is given.
(TEX)
