# Correction: The Timing Statistics of Spontaneous Calcium Release in Cardiac Myocytes

**DOI:** 10.1371/annotation/10d4ef64-c7e6-43ff-8bd7-658d47689855

**Published:** 2013-06-14

**Authors:** Mesfin Asfaw, Enric Alvarez-Lacalle, Yohannes Shiferaw

The Supporting Information files were erroneously supplied as LaTeX files. Please see Appendix S1 and Appendix S2 at the following links:
