# Supplementary material for: Correction: The Timing Statistics of Spontaneous Calcium Release in Cardiac Myocytes
Source: PLoS One. 2013 Jun 14;8(6):10.1371/annotation/10d4ef64-c7e6-43ff-8bd7-658d47689855. doi: 10.1371/annotation/10d4ef64-c7e6-43ff-8bd7-658d47689855 (PMC3683091; doi:10.1371/annotation/10d4ef64-c7e6-43ff-8bd7-658d47689855)
Supplement: Supplementary file 1 [file pone.10d4ef64-c7e6-43ff-8bd7-658d47689855.s001.pdf]

Supporting Material: Appendix S1

1 The mean first passage time for a single cluster

When the spark activation for the cluster is dictated by the closed to open transitions, the stochastic dynamics of the cluster is governed by the master equation

$$\frac{dP(n,t)}{dt} = w_+(n-1)P(n-1,t) + w_-(n+1)P(n+1,t) - \left(w_+(n) + w_-(n)\right)P(n,t) \qquad (1)$$

where $P(n,t)$ is the probability for n channels in the cluster to open at a time t. For the system dynamics governed by Eq. (1), the first passage time from n_i to n_f, with reflecting boundary conditions at origin (see the work [1]), is given by

$$T_e(n_i,n_f) = \sum_{k=n_i}^{n_f-1} \frac{1}{w_+(k)} + \sum_{k=n_i}^{n_f-2} \frac{1}{w_+(k)} \sum_{i=k+1}^{n_f-1} \prod_{j=k+1}^{i} \frac{w_-(j)}{w_+(j)} \qquad (2)$$

where $w_+(n) = k_+(N-n)(c_o+gn)^2$ and $w_-(n) = k_-n$. As described in the text we will consider the case $n_i = 0$ and $n_f = N/2$. Following Doering et al. [2] we will find an assymptotic expansion, valid for large N, for the second term in the sum above. This sum is

$$T_b(n_i,n_f) \approx \sum_{k=n_i}^{n_f-2} \frac{1}{w_+(k)} \sum_{i=k+1}^{n_f-1} \prod_{j=k+1}^{i} \frac{w_-(j)}{w_+(j)} = \sum_{k=n_i}^{n_f-2} \frac{1}{w_+(k)} \Gamma(k) \qquad (3)$$

where

$$\Gamma(k) = \sum_{i=k+1}^{n_f-1} \prod_{j=k+1}^{i} \frac{w_-(j)}{w_+(j)}. \qquad (4)$$

$\Gamma(k)$ can be rewritten as

$$\Gamma(k) = \sum_{i=k+1}^{n_f-1} e^{Nf(i)} \tag{5}$$

where

$$f(i) = \frac{1}{N}\sum_{j=k+1}^{i} \log\left(\frac{w_-(j)}{w_+(j)}\right). \tag{6}$$

Let us first define $z = j/N$ and $h(z) = 1/\rho(z) = \frac{w_-(Nz)}{w_+(Nz)}$. Via the Trapezium rule, one can rewrite Eq. (6) as

$$f(i) = \frac{\log\left(h(\frac{k+1}{N})\right) + \log\left(h(\frac{i}{N})\right)}{2N} + \int_{(k+1)/N}^{i/N} \log\left(h(z)\right) dz. \tag{7}$$

Substituting Eq. (7) in Eq. (5) and after some algebra, we get

$$\begin{aligned}\Gamma(k) &= \sqrt{h\left(\frac{k+1}{N}\right)} \exp\left(-N\phi\left(\frac{k+1}{N}\right)\right) \sum_{i=k+1}^{n_f-1} \sqrt{h\left(\frac{i}{N}\right)} \exp\left(N\phi\left(\frac{i}{N}\right)\right) \\ &\approx \sqrt{h\left(\frac{k+1}{N}\right)} \exp\left(-N\phi\left(\frac{k+1}{N}\right)\right)\chi \end{aligned} \tag{8}$$

where

$$\chi = N\int_{(k+1)/N}^{(n_f-1)/N} \sqrt{h\left(\frac{i}{N}\right)} \exp\left(N\phi\left(\frac{i}{N}\right)\right). \tag{9}$$

Here $\phi(x)$ denotes the effective potential which is given as

$$\phi(x) = \int_0^x \log\left(h(z)\right) dz = -\int_0^x \log\left(\rho(z)\right) dz. \tag{10}$$

1.1 Case 1: clusters of size $N < N_c$

In this case the fixed point at $x_o = 0$ is stable and a Ca spark occurs when the system makes a random excursion to $x_s = n_s/N$. The MFPT for the system to reach x_s starting from x_o is, to leading order, given by

$$T_b \approx \exp\left(N\left(\phi\left(\frac{n_s}{N}\right) - \phi\left(\frac{1}{N}\right)\right)\right) \approx \exp\left(N\phi\left(x_s\right)\right). \tag{11}$$

In the limit of small c_o ($c_o \approx 0$), we approximate $h(x) = 1/\left(\eta N^2(1-x)x\right)$. The effective potential (10) can be readily calculated as

$$N\phi(x_s) \approx An_s + N\log\left(1 - x_s\right) \tag{12}$$

where $A = 2 - \log\left(\rho(x_s)\right)$. Note here that since T_b is exponentially large it is safe to drop the first sum in Eq. (2) which decreases with increasing N. Thus, in this regime $T_e \approx T_b$.

1.2 Case 2: cluster of size $N_c < N < N_d$

Let us now further approximate the integral (9). Closer inspection of the effective potential (10) shows that, in this regime, $\phi(i/N)$ exhibits a maximum value at $i = m$ and hence we can impose a saddle point approximation on Eq. (9) to get

$$\chi \approx \sqrt{\frac{2\pi N h(m/N)}{\phi''(m/N)}} \exp\left(N\phi(m/N)\right). \tag{13}$$

Note here that since we are in the bistable regime the location of the maximum $m < N/2$. Substituting Eq. (8) in Eq. (3), and setting $n_i = 0$, gives

$$T_b = \chi \sum_{k=0}^{n_f - 2} \frac{1}{w_+(k)} \sqrt{h\left(\frac{k+1}{N}\right)} \exp\left(-N\phi\left(\frac{k+1}{N}\right)\right) \tag{14}$$

The last summation of Eq. (14) is dominated by the minimum of ϕ around $k = 0$. Hence let us expand ϕ around $1/N$ as

$$\phi\left(\frac{k+1}{N}\right) = \phi\left(\frac{1}{N}\right) + \phi'\left(\frac{1}{N}\right)\frac{k}{N} + ... \tag{15}$$

Plugging Eq. (15) into Eq. (14) and after some algebra, we get

$$T_b = \sqrt{\frac{2\pi N h\left(\frac{m}{N}\right)}{\phi''\left(\frac{m}{N}\right)}} \exp\left(N\left(\phi\left(\frac{m}{N}\right) - \phi\left(\frac{1}{N}\right)\right)\right) \frac{h\left(\frac{1}{N}\right)^{\frac{3}{2}}}{w_+(0)\left(h\left(\frac{1}{N}\right) - 1\right)} \tag{16}$$

where $h(x) = 1/\left(\eta N^2(1-x)x\right)$.

The fixed point related to $h(x)$ is given by

$$x_{1,2} = \frac{1}{2}\left(1 \pm \sqrt{1 - \frac{4}{N^2\eta}}\right). \tag{17}$$

Let us now evaluate the prefactor in Eq. (16). We find

$$w_+(0) = k_+ c_0^2 N, \tag{18}$$

$$h\left(\frac{m}{N}\right) = 1, \tag{19}$$

$$\frac{h(\frac{1}{N})^{\frac{3}{2}}}{h(\frac{1}{N}) - 1} = \sqrt{\frac{1}{N\eta}} \tag{20}$$

and

$$\phi''(x_1) = -N^2\eta. \tag{21}$$

Let us now apply a binomial expansion on Eq. (17) in order to get

$$x_1 = \frac{1}{(\eta N^2)} + \frac{1}{(\eta N^2)^2} + \frac{2}{(\eta N^2)^3}. \tag{22}$$

As approximation, let us take

$$h(x) \approx \frac{1}{N^2 \eta x} \tag{23}$$

and evaluate

$$\begin{aligned} \phi(m/N) - \phi(1/N) &= \int_{1/N}^{x_1} \log\left(h(x)\right) dx \\ &= \frac{1}{(\eta N^2)} + \frac{1}{(\eta N^2)^2} - \frac{1}{N} + \frac{\log(\eta N)}{N}. \end{aligned} \tag{24}$$

Finally, substituting Eqs. (18-21) and Eq. (24)in Eq. (16) leads to

$$T_b = \frac{\sqrt{2\pi}}{k_+ c_0^2 N} \exp\left(\frac{1}{\eta N} + \frac{1}{(\eta N)^2 N} - 1\right). \tag{25}$$

We note here that in this regime we have $N < N_d = 1/\eta$, so that the exponent in the expression above is large since $1/\eta N >> 1$. Thus, the first sum in Eq. (2), which decreases with increasing N, is negligible and $T_e \approx T_b$.

1.3 Case 3: cluster of size $N > N_d$.

For large clusters with channels $N > N_d = 1/\eta$ we note that the exponential term in Eq. (25) approaches a constant value. Thus, our assymptotic estimate for T_b is dominated by the prefactor, which is proportional to $\sim 1/(Nk_+c_o^2)$. However, we should note that the assymptotic approximation itself breaks down in this scenario since the fixed point at x_1 is small i.e. we are in the discrete bistable regime. In this case, as discussed in the text, the MFPT can be approximated as the MFPT to the first few channel openings. Specifically, if we consider the MFPT to two open channels ($n_i = 0, n_f = 2$) we get

$$T_e \approx \frac{1}{w_+(0)} + \frac{1}{w_+(1)} + \frac{1}{w_+(0)}\frac{w_-(1)}{w_+(1)}, \tag{26}$$

which is

$$T_e \approx \frac{1}{k_+Nc_0^2} + \frac{1}{k_+(N-1)(c_o+g)^2} + \frac{1}{k_+Nc_o^2}\frac{k_-}{k_+(N-1)(c_o+g)^2}. \tag{27}$$

Now, since our physiological parameters require that $c_o << g$ and $N >> 1$ we can make the approximation that $c_o + g \approx g$ and $N - 1 \approx N$, so that

$$T_e \approx \frac{1}{k_+Nc_0^2} + \frac{1}{k_+Ng^2} + \frac{1}{k_+Nc_o^2}\left(\frac{1}{\eta N}\right), \tag{28}$$

$$T_e \approx \frac{1}{k_+Nc_0^2}\left(1 + \frac{1}{\eta N} + \epsilon^2\right), \tag{29}$$

where $\epsilon = c_o/g$. For the range of physiological parameters we note that $\epsilon^2 << 1/\eta N$, so that we have

$$T_e \approx \frac{1}{k_+Nc_0^2}\left(1 + \frac{1}{\eta N}\right). \tag{30}$$

1.4 Case 4: clusters of size $N >> N_d$

For $N >> N_d$ the second term in Eq. (30) is small and we have

$$T_e \approx \frac{1}{k_+Nc_0^2}. \tag{31}$$

which is simply the MFPT to one open channel in the cluster.

References

[1] P. Pury, M. Caceres, Mean first-passage and residence times of random walks on asymmetric disordered chains, J. Phys. A: Math. Gen., **36**, 2695 (2003).

[2] C. R. Doering, K. V. Sargsyan and L. M Sander, Extinction Times for Birth-Death Processes: Exact Results, Continuum Asymptotics, and the Failure of the Fokker-Planck Approximation, SIAM Journal of Multiscale Modeling and Simulations **3** (2), 283-299 (2005).
