# Supplementary material for: Correction: The Timing Statistics of Spontaneous Calcium Release in Cardiac Myocytes
Source: PLoS One. 2013 Jun 14;8(6):10.1371/annotation/10d4ef64-c7e6-43ff-8bd7-658d47689855. doi: 10.1371/annotation/10d4ef64-c7e6-43ff-8bd7-658d47689855 (PMC3683091; doi:10.1371/annotation/10d4ef64-c7e6-43ff-8bd7-658d47689855)
Supplement: Supplementary file 2 [file pone.10d4ef64-c7e6-43ff-8bd7-658d47689855.s002.pdf]

# Supporting Material: Appendix S2

## 1 The mean first passage time for time varying SR load

### 1.1 MFPT for a single cell

In the case of time varying SR load, as discussed in the manuscript, the first passage distribution (FPD) which is denoted by $P(t)$ is given as

$$P(t) = \left(\frac{1}{T_M}\right)\Theta(t-t_c)\exp\left(-\frac{t-t_c}{T_M}\right) \tag{1}$$

where

$$T_M = \frac{\beta}{k_+ c_o^2 M} \tag{2}$$

and $t_c$ is refractory duration time. The MFPT is evaluated as

$$\begin{aligned} T &= \int_0^\infty tP(t)dt = \int_{t_c}^\infty \left(\frac{t}{T_M}\right)\exp\left(-\frac{t-t_c}{T_M}\right)dt \\ &= t_c + T_M. \end{aligned} \tag{3}$$

### 1.2 The timing distribution of SCR across an ensemble of cells

Let us now compute the timing distribution of SCR in tissue. Given $P_i(t)$ as the FPD for $i^{th}$ cell (see Eq. (32)), the number of cells in a tissue in which SR occurs in the time interval $[t, t+dt]$ is given by $KP_R(t)dt$, where the distribution is then given by

$$P_R(t) = \frac{1}{K}\sum_{i=1}^{K} P_i(t) \tag{4}$$

The MFPT $T_K$ is then given by

$$\begin{aligned}
T_K &= \frac{1}{K}\sum_{i=1}^{K}\int_0^{\infty} tP_i(t)dt \\
&= \frac{1}{K}\sum_{i=1}^{K}\left(t_c^i + T_M^i\right) \\
&= \langle t_c\rangle + \langle T_M\rangle . \qquad (5)
\end{aligned}$$

Let us now compute the standard deviation of $P_R(t)$ which is defined as

$$\begin{aligned}
\sigma^2 &= \int_0^{\infty} t^2 P_R(t)dt - \left(\int_0^{\infty} tP_R(t)dt\right)^2 \\
&= \int_0^{\infty} t^2 P_R(t)dt - T_K^2 \\
&= \int_0^{\infty} t^2 P_R(t)dt - \left(\langle t_c\rangle^2 + 2\langle t_c\rangle\langle T_M\rangle + \langle T_M\rangle^2\right). \qquad (6)
\end{aligned}$$

Now what remains is to compute the first integral of Eq. (34)

$$\begin{aligned}
\int_0^{\infty} t^2 P_R(t)dt &= \frac{1}{K}\sum_{i=1}^{K}\int_{t_c}^{\infty}\frac{t^2}{T_M^i}\exp\left(-\frac{t-t_c^i}{T_M^i}\right)dt \\
&= \left\langle t_c^2\right\rangle + 2\langle t_c T_M\rangle + 2\left\langle T_M^2\right\rangle . \qquad (7)
\end{aligned}$$

Substituting Eq. (38) in Eq. (37) yields

$$\sigma^2 = \left\langle t_c^2\right\rangle - \langle t_c\rangle^2 + 2\left\langle T_M^2\right\rangle - \langle T_M\rangle^2 + 2\langle t_c T_M\rangle - 2\langle t_c\rangle\langle T_M\rangle . \qquad (8)$$

Note that the last two terms are just $2Cov(t_c, T_M)$ which is zero since $t_c$ and $T_M$ are independent. Hence, Eq. (37) simplifies to

$$\sigma^2 = \sigma_c^2 + \sigma_M^2 \qquad (9)$$

where

$$\sigma_c^2 = \left\langle t_c^2\right\rangle - \langle t_c\rangle^2 \qquad (10)$$

and

$$\sigma_M^2 = 2\left\langle T_M^2\right\rangle - \langle T_M\rangle^2 . \qquad (11)$$
